# Zero-Heat-Flux Thermometry for Non-Invasive Measurement of Core Body Temperature in Pigs

**DOI:** 10.1371/journal.pone.0150759

**Published:** 2016-03-03

**Authors:** Maria Guschlbauer, Alexandra C. Maul, Xiaowei Yan, Holger Herff, Thorsten Annecke, Anja Sterner-Kock, Bernd W. Böttiger, Daniel C. Schroeder

**Affiliations:** 1 Center for Experimental Medicine, University Hospital of Cologne, Cologne, Germany; 2 Department of Anaesthesiology and Intensive Care Medicine, University Hospital of Cologne, Cologne, Germany; University of Minnesota, UNITED STATES

## Abstract

Hypothermia is a severe, unpleasant side effect during general anesthesia. Thus, temperature surveillance is a prerequisite in general anesthesia settings during experimental surgeries. The gold standard to measure the core body temperature (T_core_) is placement of a Swan-Ganz catheter in the pulmonary artery, which is a highly invasive procedure. Therefore, T_core_ is commonly examined in the urine bladder and rectum. However, these procedures are known for their inaccuracy and delayed record of temperatures. Zero-heat-flux (ZHF) thermometry is an alternative, non-invasive method quantifying T_core_ in human patients by applying a thermosensoric patch to the lateral forehead. Since the porcine cranial anatomy is different to the human’s, the optimal location of the patch remains unclear to date. The aim was to compare three different patch locations of ZHF thermometry in a porcine hypothermia model. Hypothermia (33.0°C T_core_) was conducted in 11 anesthetized female pigs (26-30kg). T_core_ was measured continuously by an invasive Swan-Ganz catheter in the pulmonary artery (T_pulm_). A ZHF thermometry device was mounted on three different defined locations. The smallest average difference between T_pulm_ and T_ZHF_ during stable temperatures was 0.21 ± 0.16°C at location A, where the patch was placed directly behind the eye. Also during rapidly changing temperatures location A showed the smallest bias with 0.48 ± 0.29°C. Location A provided the most reliable data for T_core_. Therefore, the ZHF thermometry patch should be placed directly behind the left temporal corner of the eye to provide a non-invasive method for accurate measurement of T_core_ in pigs.

## Introduction

Pigs are commonly used as experimental animal models to mirror human conditions [1–5]. Therefore, they often undergo general anesthesia and are prone to develop a hypothermic status, known as perioperative hypothermia [3, 6–10]. Anesthesia-induced impairment of thermoregulatory control, redistribution of core body heat to the periphery and reduction of metabolic energy production are considered to be responsible for perioperative hypothermia [11–14]. Various consequences such as coagulopathy [7, 15–17], imbalances in the electrolyte metabolism, increased hematocrit, tubular necrosis, shivering [18–21] as well as higher incidence of wound infections and healing have been described [7, 22–24]. To avoid the impact of perioperative hypothermia on experimental data, accurate measurement of core body temperature (T_core_) is a prerequisite during general anesthesia [7, 11, 25–29]. The gold standard to record T_core_ is to measure the blood temperature in the pulmonary artery (T_pulm_) using a Swan-Ganz catheter [6, 28, 30–32]. However, placement of a Swan-Ganz catheter is highly invasive and thus not always suitable in porcine experimental settings.

Peripheral measurement sites, such as the temperatures in urinary bladder and rectum, are commonly used to continuously monitor T_core_. Albeit, peripheral temperature measurement sites show a time delay compared to pulmonary artery temperature during rapidly changing temperatures resulting in a misinterpretation of T_core_ [33–36].

In human medicine a non-invasive method to accurately evaluate core body temperature is the Zero-heat-flux (ZHF) technology, first described in 1973 [37]. An insulator patch applied to the lateral forehead and covered by an electric heater is used to stop surface convection, creating an isothermic tunnel from the core body to the skin surface. As soon as heater- and skin-temperatures are equal, the subdermal temperature can be measured approximately 1 to 2 cm below the skin surface. In well-perfused parts of the body, tissue temperature below the skin surface approximates core body temperature [7, 26, 29, 37, 38].

To date, ZHF technology has not been evaluated in pigs. As the porcine cranial anatomy differs from the human structures, the appropriate patch location on the porcine head is unknown. The aim of the present study was to compare three different ZHF patch locations on the porcine forehead to evaluate whether ZHF technology is feasible in pigs. Therefore, the ZHF device was tested during an experimentally induced porcine model of mild therapeutic hypothermia. It was hypothesized that in pigs the ZHF device, placed on a defined location at the forehead, serves as a reliable non-invasive method to evaluate T_core_ corresponding to pulmonary artery temperature.

## Material and Methods

### Animals

11 female crossbred growing pigs (Landrace x Pietrain) weighing 29.1 ± 1.4 kg underwent a hypothermia protocol under general anesthesia. All experimental procedures were ethically approved by the governmental authority responsible for animal welfare in the state of North Rhine-Westphalia (Landesamt für Natur, Umwelt und Verbraucherschutz Nordrhein-Westfalen, Germany). All procedures were in accordance with the German Laws for Animal Protection. Animal care and use was performed by qualified staff members, supervised by a veterinarian, and all facilities and transportation procedures comply with current legal requirements. Pigs were purchased from a local breeding farm (Kalkar, Germany). Animals were allowed to acclimatize for at least 10 days before interventions started. The pigs were group housed in straw bedded 9.3 m^2^ boxes (groups of 2 to 5 animals) in the Centre for Experimental Medicine at the University Hospital of Cologne. They were fed a standard diet (900 g/animal/day, Universal Mast, RWZ, Cologne, Germany) and had free access to water. To satisfy exploring behavior, enrichment was provided and hay was offered daily. Photoperiods were 12:12 hours light:dark and ambient temperature was maintained at 20±1°C. At the end of the intervention pigs were euthanized using an overdose of pentobarbital-sodium (80mg/kg; Euthadorm, CP Pharma, Burgdorf, Germany) injected intravenously during deep anesthesia.

### Anesthesia and analgesia

Pigs were fasted for 12 hours prior to the start of the interventions while water was always accessible. Before induction of anesthesia, the animals were separated from the group, with remaining visual contact. The pigs received an intramuscular injection of azaparone (2 mg/kg body weight; Stresnil, Janssen, Neuss, Germany), ketamine (20 mg/kg body weight; Ketavet 100, Pfizer, Berlin, Germany) and atropine (0.02 mg/kg body weight, Atropin, Braun, Melsungen, Germany) for premedication. During transport to the intervention room, oxygen was administered via face mask. Pigs were bedded in a supine position. By use of a 20 gauge catheter (Vasovet, Braun Melsungen, Melsungen, Germany) in the lateral auricular vein, a bolus of propofol (Propofol 2% MCT, Fresenius, Bad Homburg, Germany) was administered prior to endotracheal intubation using an endotracheal tube (6–6.5 ID mm, Teleflex Medical, Kernen, Germany). Ventilation was performed using a volume-controlled ventilator setting (Fabius GS, Dräger, Lübeck, Germany) of 8 ml per kilogram bodyweight tidal volume to obtain normocapnia with a PaCO_2_ of 35 mmHg to 45 mmHg, a FIO_2_ = 0.25 and a positive end-expiratory pressure of 5 mmHg. Total intravenous anesthesia (TIVA) using a combination of propofol (5–7 mg/kg/h i.v.), midazolam (1.2 mg/kg/h i.v.; Midazolam, Rotexmedica, Trittau, Germany) and fentanyl (12–15 μg/kg/h i.v.; Fentanyl, Rotexmedica, Trittau, Germany) was applied to maintain anesthesia. Fluid management was performed using 37°C preheated Ringer’s solution (Ringerlösung Fresenius, Fresenius Kabi, Bad Homburg, Germany) 7 to 10 ml/kg per hour, dependent on the circulatory situation. A standard lead II electrocardiogram was used to monitor cardiac rhythm (Philips Medizinsysteme, Böblingen, Germany).

### Preparation, catheters and temperature measurement

**Fig 1:**
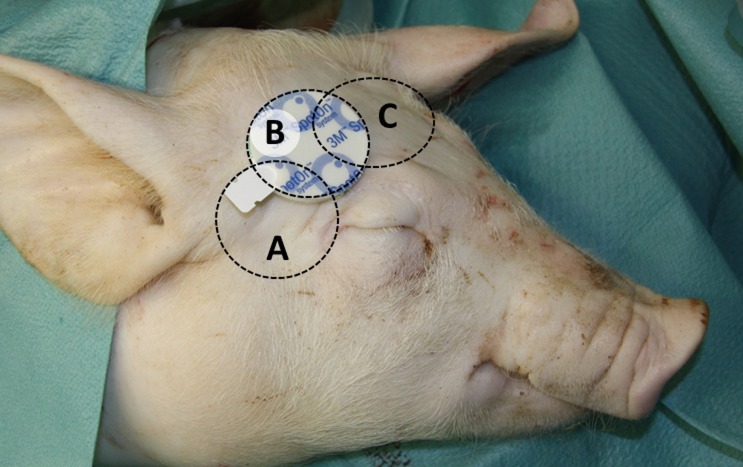
Example of different ZHF patch locations on the head of a euthanized pig. The patch is located at position B (obliquely above the eye), circles indicate placement at location A (directly behind the temporal corner of the eye) and C (centered on the forehead).

When the state of surgical tolerance was reached, a 14 gauge saline-filled catheter (Arrow International, Reading, USA) was inserted into the right *Vena femoralis* for TIVA and fluid administration. The right *Arteria femoralis* was catheterized (Arterial leadercath, Vygon, Ecouen, France) to record arterial blood pressure using a transducer that was aligned at the level of the right atrium (Philips M1097A, Philips Medizinsysteme, Böblingen, Germany). A Swan-Ganz catheter (5 French, Arrow International, Reading, USA) was inserted via the right internal jugular vein into the pulmonary artery in order to measure pulmonary artery temperature. A urine catheter (12 Ch, Balloon Catheter, Teleflex Medical, Kernen, Germany) was placed in the bladder via median laparotomy. Spontaneous cooling of the pigs before starting the experiment was prevented by covering the animals with heating-blankets (Bairhugger, 3M, Neuss, Germany). A 3M SpotOn patch (3M^TM^ SpotOn^TM^, 3M, Neuss, Germany) was stuck to the shaved skin on three different, defined localizations (Fig 1): A: directly behind the left temporal corner of the eye (4 pigs), B: obliquely above the eye on the forehead, this position is equivalent to human application of 3M SpotOn (3 pigs), C: central on the forehead (4 pigs).

Thereafter the Zero-heat-flux temperature (T_ZHF_) device was connected to the patches to record temperature. A cooling system (Variotherm 555, Hirtz & coKG, Cologne, Germany) was applied intraoesophageally. Mild therapeutic hypothermia (33°C) was conducted by an automatic feedback cooling system. The cooling device continuously registered T_pulm_ and automatically adjusted the cooling-temperature to the pre-settings of the study protocol.

### Experimental Design

Pigs were cooled from T_pulm_ = 37.7 ± 0.6°C to 33°C as fast as possible (= cooling phase). Thereafter, T_pulm_ was kept at 33°C (= maintaining phase) for 1 hour. During this process T_ZHF_ and T_pulm_ were recorded every 5 minutes to determine the precision of the ZHF device in measuring core body temperature compared to T_pulm_. Altogether the study included 363 paired temperature measurements of T_ZHF_ and T_pulm_ that were used for statistical analysis. n = 130 paired measurements were recorded at ZHF location A in four pigs, thereof n = 78 paired measurements during the cooling phase and n = 52 during the maintaining phase. At ZHF location B altogether n = 98 paired measurements were recorded in three pigs, thereof n = 59 during the cooling phase and n = 39 during the maintaining phase. At ZHF location C in total n = 135 paired measurements were recorded in four pigs, thereof n = 83 were measured during the cooling phase and n = 52 during the maintaining phase. The differences in the number of paired measurements per animal in the cooling phase resulted from the different durations of this specific phase.

### Statistics

Statistical analysis was performed using the Bland-Altman plot [39, 40] to calculate the bias and 95% limit of agreement (GraphPad Prism Version 6.0, GraphPad Software, La Jolla, California). Each dot in the Bland Altman plot represents the results of one temperature pair of T_pulm_ and T_ZHF_ in one animal at time t. The paired Student’s t-tests were performed with Stata (Stata 13.1, DatacorpLP, College Station, USA). Values are expressed as mean ± SD. P values less than 0.05 were set as significant. Starting point of the maintaining phase was defined as the time when the difference between two following T_ZHF_ values was zero. The bias of T_ZHF_ compared to T_pulm_ in the cooling phase was statistically decomposed into two factors: a *level effect* caused by the intrinsic ZHF device bias and a *time effect* that caused the delay of T_ZHF_ during the rapidly changing temperatures in the cooling phase.

## Results

**Fig 2:**
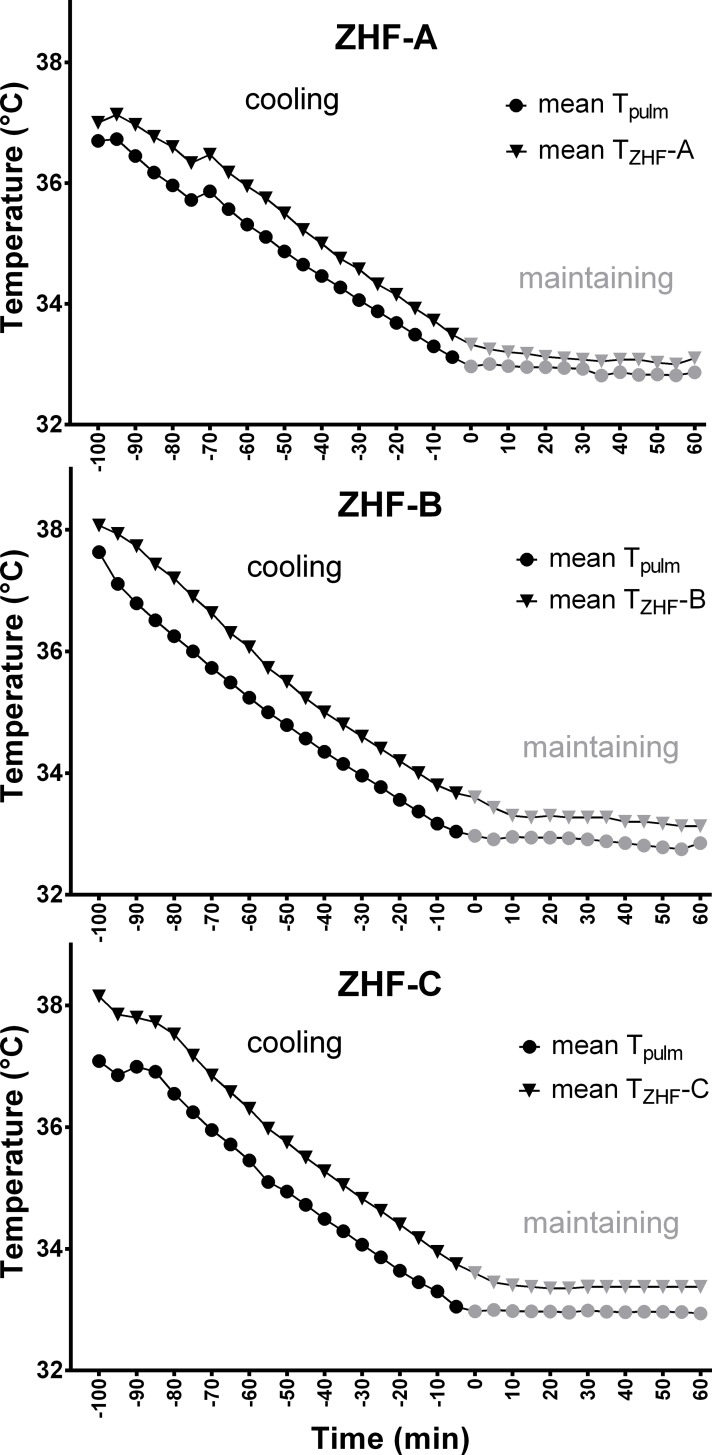
**Pattern of mean temperatures during cooling (black) and maintaining phase (grey) at ZHF locations A (4 pigs), B (3 pigs) and C (4 pigs).** Data points represent the mean temperature (T_ZHF_ or T_pulm_) of the respective group for each time point (every 5 minutes). Number of paired measurements at ZHF location A: n = 130, at ZHF location B: n = 98, at ZHF location C: n = 135. T_pulm_ = blood temperature in the pulmonary artery; T_ZHF_ = temperature measured by the ZHF device.

Mean cooling rate was 2.8 ± 0.46°C/h. Goal temperature of T_pulm_ = 33°C was attained after 100 ± 17 minutes. An overview of the temperature patterns are given in Fig 2.

**Fig 3:**
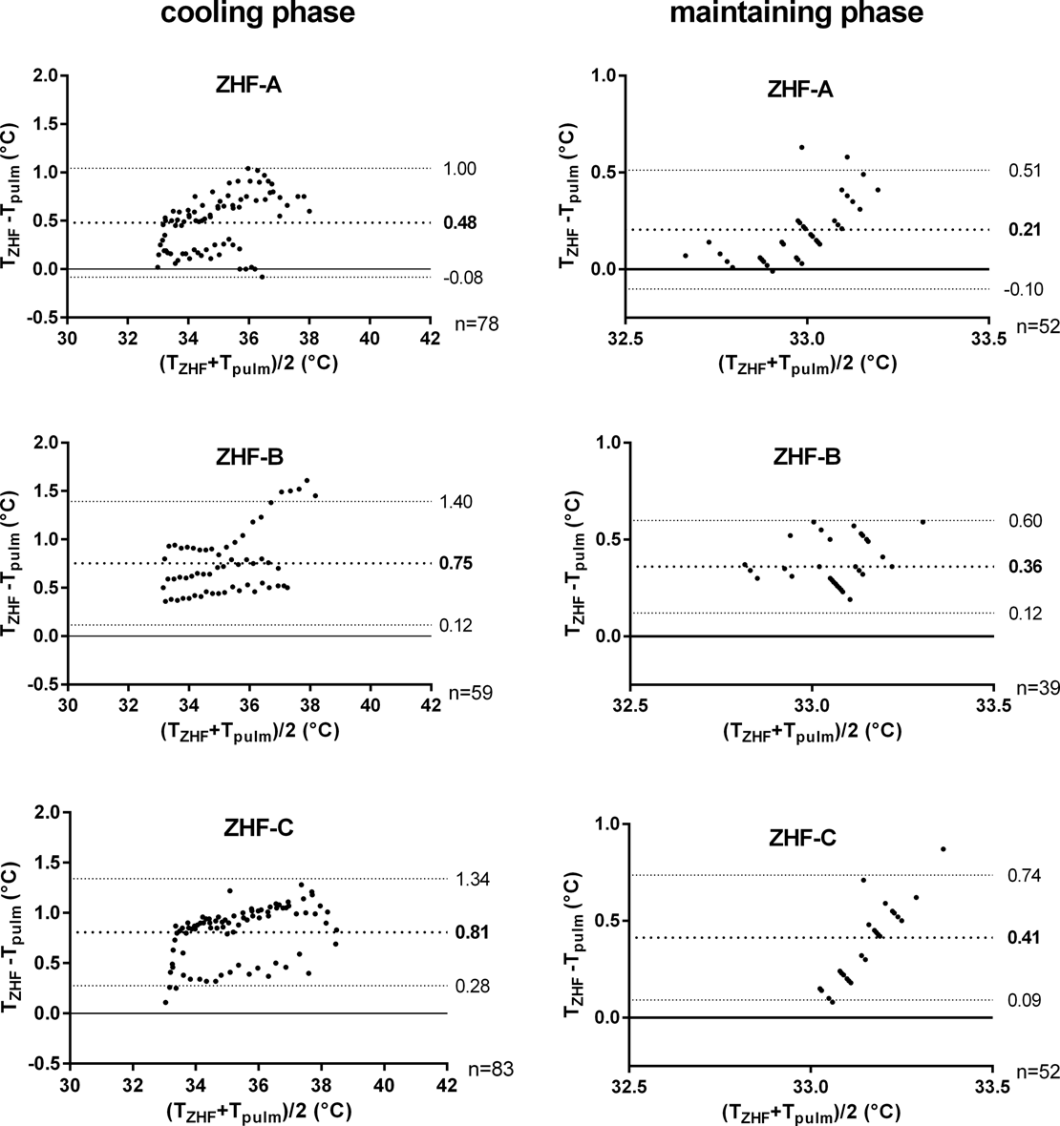
**Bland Altman plots comparing T**_**pulm**_ **and T**_**ZHF**_ **for ZHF location A (4 pigs), B (3 pigs) and C (4 pigs) during cooling and maintaining phase.** T_pulm_ = blood temperature in the pulmonary artery, T_ZHF_ = temperature measured by the ZHF device.

T_pulm_ differs significantly from T_ZHF_ at all three locations during cooling and maintaining phase (p<0.001). Results of Bland Altman plots for cooling and maintaining phase are shown in Fig 3 and Table 1.

**Table 1:** Bias of T_ZHF_ and T_pulm_ for each ZHF location (A, B, C) in cooling phase, maintaining phase and the time effect.

Table 1	cooling phase	maintaining phase (≙ level effect)	time effect during cooling phase (= bias of cooling phase–level effect)
**bias-A**	**0.48** ± 0.29	**0.21** ± 0.16^§^	**0.27** ± 0.28
**bias-B**	**0.75** ± 0.33***	**0.36** ± 0.12***^/^^§^	**0.39** ± 0.33*
**bias-C**	**0.80** ± 0.27***	**0.41** ± 0.16***^/^^§^	**0.39** ± 0.27***

Bias = mean difference between T_ZHF_ and T_pulm_. Data are shown as mean difference ± SD (°C)

*** = significantly different to bias-A (p<0.001)

* = significantly different to bias-A (p<0.05)

§ = significantly different to the bias in the corresponding cooling phase (p<0.001)

As the bias during the cooling phase was significantly higher compared to the maintaining phase at all three locations (A, B, C) (Table 1), the bias was statistically split into two effects: a level effect, caused by the ZHF device that exists in both phases, and a time effect, present only during rapidly changing temperatures in the cooling phase. **Level effect:** both temperature measuring methods revealed constant temperatures during the maintaining phase (Fig 2). Therefore, the bias during the maintaining phase was equalized as the intrinsic *level effect* of the ZHF device. Results are shown in Table 1. **Time effect:** T_ZHF_ values of the cooling phase were adjusted for the level effect (= T_ZHF-adj_) to quantify the time effect on the bias in the cooling phase. Data are shown in Table 1. The delay-duration of T_ZHF_ compared to T_pulm_ was calculated by comparing T_pulm_ temperatures (at timepoint t_0_) with T_zhf-adj_ temperatures, which were both recorded at time points 5 (t_0+5_), 10 (t_0+10_) and 15 (t_0+15_) minutes after t_0_ (Table 2).

**Table 2:** Time delay of T_ZHF_ compared to T_pulm_ during the cooling phase.

	T_pulm_ at t_0_ & T_ZHF-adj_ at t_0+5_	T_pulm_ at t_0_ & T_ZHF-adj_ at t_0+10_	T_pulm_ at t_0_ & T_ZHF-adj_ at t_0+15_
**bias-A**	**0.08**±0.23**	**-0.11**±0.18^§^	**-0.30**±0.17^§^
**bias-B**	**0.17**±0.29***	**-0.05**±0.27	**-0.26**±0.26^§^
**bias-C**	**0.16**±0.26***	**-0.07**±0.27^#^	**-0.28**±0.30^§^

Bias was calculated comparing T_pulm_ at t_0_ and T_ZHF-adj_ at 5, 10 and 15 minutes after t_0_ (t_0_+5, t_0_+10, t_0_+15) for ZHF location A, B and C. Data are shown as mean difference ± SD (°C), bias = mean difference between T_zhf-adj_ and T_pulm_

** = T_ZHF-adj_ is significantly higher than T_pulm_ (p<0.01)

*** = T_ZHF-adj_ is significantly higher than T_pulm_ (p<0.001)

^§^ = T_ZHF-adj_ is significantly lower than T_pulm_ (p<0.001)

^#^ = T_ZHF-adj_ is significantly lower than T_pulm_ (p<0.05)

T_ZHF_-A showed a time delay between 5 and 10 minutes compared to T_pulm_. At location B the time delay was exactly 10 minutes because T_ZHF-adj_-B at t_0+10_ did not significantly differ from T_pulm_ at t_0_ (p>0.05). T_ZHF-adj_-C at t_0+10_ is significantly lower than T_pulm_ at t_0_ (p<0.05) indicating a time delay between 5 and 10 minutes.

## Discussion

In the present study it could be demonstrated for the first time that Zero-heat-flux technology for temperature measurement is applicable in pigs. Three different locations to place thermosensoric patches for the non-invasive Zero-heat-flux temperature measurement were compared, using pulmonary artery temperature for comparison.

Accurate management of core body temperature in surgical settings under general anesthesia is indispensable in humans as well as in pigs in order to prevent perioperative hypothermia. Furthermore, in experimental settings, where normothermia is desirable, identification of a rapidly changing T_core_ is necessary in order to prevent perioperative hypothermia or malignant hyperthermia. Hence, reliable methods for temperature surveillance are a prerequisite for different surgical interrogations in the pig [3, 25, 28, 36, 41].

Common, non-invasive methods to measure core body temperature in pigs during anesthesia are placement of a thermosensoric rectal probe or a urinary bladder catheter for temperature measurement. Those peripheral sites do not reliably display fast changes of the core body temperature in humans and pigs. Literature revealed that both, urinary and rectal temperature, showed bias values >0.5°C compared to T_core_. Additionally, urinary bladder temperature may be misinterpreted due to decreased urine production [25, 26, 30, 32–34, 36]. Musk et al. (2015) compared porcine rectal to oesophageal temperatures during small surgery procedures. They showed a bias of 0.69°C, with 95% limits of agreement of -1.18 to 2.57°C taken from Bland-Altman analysis. The high bias and the wide range of the limits of agreement imply that rectal temperature is unsuitable for measuring core body temperature [34].

The ZHF device in human application accomplished a bias of -0.23°C with 95% limits of agreement of -1.05 and +0.59°C, compared to pulmonary artery temperature [29]. In accordance to the results of Eshraghi et al. (2014), in the present study the porcine application of the ZHF device at location A showed a bias of 0.21 ± 0.16°C in the maintaining phase. Both locations, B and C, revealed a significantly higher bias than location A (Table 1). Nevertheless, considering a maximal accepted difference between two comparable temperature measuring methods of 0.5°C [11, 29, 42] all ZHF locations show a clinically acceptable bias in the maintaining phase (Table 1). In summary, the ZHF device at location A is applicable for a reliable measurement of constant T_core_ in pigs. During the cooling phase the bias was significantly higher than in the maintaining phase at all three locations (A, B, C) (p<0.001, Table 1). Thus, an influence of fast changing temperatures on the reliability of T_ZHF_ was supposed. Similar phenomena have already been described before [1, 33, 36]. In a study of Krizanac et al. (2010) temperatures measured by tracheal temperature probes were compared to the pulmonary artery temperature. Fast cooling resulted in a significantly higher bias than slow cooling, implicating a delay of tracheal compared to pulmonary artery temperature during rapidly changing temperatures [1]. During the cooling phase, the time effect was quantified (Table 1) and the delay of T_ZHF_ compared to T_pulm_ was evaluated as 5 to 10 minutes for location A and C and exactly 10 minutes for location B. Bias during cooling at location A was 0.48 ± 0.29°C. Though a time delay of 5 to 10 minutes was present during forced cooling, the deviation of T_ZHF_ compared to T_pulm_ was in compliance with the clinically acceptable bias range of <0.5°C [11, 29, 42]. Both, location B and C, included significantly higher bias values for the cooling phase than location A (Table 1), and were therefore assessed as not convenient locations to monitor a precise temperature course compared to location A.

The setting of mild therapeutic hypothermia was chosen to examine the reliable functionality of the ZHF device during rapidly decreasing and constant temperatures. After the cooling phase, temperature was constantly maintained at 33°C, which is far below the physiological core body temperature of pigs during general anesthesia. At 33°C the ZHF device reliably displayed core body temperature. Therefore, it was extrapolated that the device is able to display a precise temperature monitoring also in constant physiological ranges, which has to be proven in further studies.

To conclude, the ZHF device placed at location A, directly behind the lateral eye angle, provides the most accurate display of core body temperature in pigs. Thus, under clinical aspects, the device is judged as applicable as a non-invasive method for porcine T_core_ measurement in experimental settings under general anesthesia.
